# “Values That Vanish into Thin Air”: Nurses' Experience of Ethical Values in Their Daily Work

**DOI:** 10.1155/2013/939153

**Published:** 2013-08-19

**Authors:** Gro Bentzen, Anita Harsvik, Berit Støre Brinchmann

**Affiliations:** ^1^University of Nordland, 8049 Bodø, Norway; ^2^Nordland Hospital, 8092 Bodø, Norway; ^3^University of Stavanger, 4046 Stavanger, Norway

## Abstract

The objective of this study was to examine how nurses experience ethical values as they are expressed in daily practice in a Norwegian hospital. A growing focus in Western healthcare on effectiveness, production, and retrenchment has an influence on professional nursing standards and nursing values. Lack of resources and subsequent ethically difficult prioritizations imply a strain on nurses. This study is qualitative. Data collection was carried out by conducting 4 focus group interviews. The data was analyzed using content analysis. The results are presented in two main themes: (1) values and reflection are important for the nurses; (2) time pressure and nursing frustrations in daily work. The results demonstrate that nurses believe the ethical values to be of crucial importance for the quality of nursing; however, the ethical values are often repressed in daily practice. This results in feeling of frustration, fatigue, and guilty conscience for the nurses. There is a need for changes in the system which could contribute to the development of a caring culture that would take care of both patients and nurses. In an endeavour to reach this goal, one could apply caritative leadership theory, which is grounded on the caritas motive, human love, and mercy.

## 1. Introduction

Western health services are characterized by increasing demands for effectiveness, production, and financial profit and economizing [1]. This has an impact on professional standards and clinical nursing values and constitutes a source of job-related stress and unrest for the nurses [2]. Norwegian studies from hospitals and local health services show that lack of resources and heavy work pressure lead to ethically difficult prioritizations and a lowering of nursing standards [3]. Vital medical needs are prioritized, while psychosocial needs are not adequately met [4]. Clinicians find themselves being disloyal to professional ideals and expectations [5]. European studies demonstrate that professional ethical codes become unattainable ideals for many nurses due to the lack of resources and heightened effectiveness in the healthcare environment [6]. Lack of resources and the lowering of priorities cause considerable strain on healthcare professionals both in hospitals and the local health services [4]. This strain can be understood as moral distress, a concept associated with the ethical dimension in practice and with issues related to difficulties in practice in terms of maintaining professional values, responsibility, and duties [7–11]. Moral distress, even though it is understood differently in different studies, has shown to have negative consequences and contributes to emotional discomfort, such as, for example, anger, frustration, withdrawal, insecurity or poor quality in patient care, reduced job satisfaction, and occupational fatigue [11–15].

We took our point of departure in the national core values in the Norwegian specialist health services: respect, quality, and safety. The Norwegian national core values for the specialist health services were introduced with the health reform in 2002. We wanted to explore whether these values after ten years are present and of importance in nurses' daily practice and how this affects the nurses' daily practice and professional wellbeing. The object of this study was to examine nurses' experience of how ethical values are expressed in daily practice in a Norwegian hospital where health reforms have been introduced as well as market thinking.

## 2. Materials and Methods

### 2.1. Participants

The collection of data was carried out by conducting focus group interviews with a total of 20 nurses from different somatic and psychiatric bed units in a medium large hospital in Norway.

The participants consisted of 13 special nurses and 7 nurses. In terms of gender, there were 18 women and 2 men. The ages of the participants were from 27 to 60 years, with 2 to 38 years of experience as nurses, the majority around 20 years. The request for participants for the study was sent to the administration in each clinic, and the participants were then selected and asked by their immediate superior if they were willing to take part in the study. The selection of participants was convenient as the request to participate was made to all the nurses in the units without any criteria due to, for example, gender or work experience. 

### 2.2. Data Collection and Analysis

#### 2.2.1. Focus Groups

Focus groups are a qualitative method where a group of people with certain common qualities are gathered together to discuss a given, well-defined topic, in permissive and nonthreatening surroundings. Focus groups should be informal and the questions should be direct, comfortable, and simple but not guiding [16]. One of the foremost advantages of focus groups is the interaction between the participants which may generate more profound and richer data than individual interviews [17].

Four focus group interviews were carried out with moderator and assistant moderator [16]. Three interviews were conducted with nurses from different somatic units and one interview with nurses from different psychiatric units. After three interviews in the somatic units, we reached a point of saturation in the data and decided to conduct a last interview with participants from psychiatric units to look for comparable results. All authors acted as moderators or assistant moderator in one or several interviews. The second author was moderator during three of the interviews, and this provided both continuity and experience in the moderator role [18]. The interviews lasted 90 minutes, they were tape recorded, and ongoing notes were taken by the assistant moderator. The focus groups took place in the hospital's meeting room during the participants' working hours. A semistructured topic guide ensured that the same key topic was under discussion in the different focus groups [16]. The topic guide's starting point was the national core values in the Norwegian specialist health service: quality, safety, and respect, and how these values were expressed or challenged in everyday work. Other topics were whether the core values or other ethical values functioned as guidance in daily practice and whether reflection about ethics and values took place in the daily work. We also asked who were responsible for compliance with the core values. The interviews were carried out in the period from December 2010 to November 2011.

#### 2.2.2. Data Analysis

The aim of the study was to explore attitudes, thoughts, feelings, experiences, and knowledge among nurses about core values and other ethical values. The method must be adapted to the research question and thus is a qualitative method chosen [19, 20]. 

The analysis is inspired by qualitative content analysis [21, 22]. The main key to content analysis is that the words in the text are classified into smaller content categories [22]. 

We experienced that the participants were greatly committed and candid in the focus groups, with the result that the material obtained was highly informative and comprehensive. In the analysis we emphasized the findings that generated most engagement and discussion among the participants.

The analysis started after each interview with listening to the tape and making a verbatim transcription of each interview. At first, all the interviews were separately analyzed. Then the results from the three interviews from the somatic units were considered across and analyzed as a whole. The interview from the psychiatric units was separately analyzed similarly to the three others, and one looked for comparable results. 

The transcriptions were read through several times by each one of us. We chose to consider the manifest content, that is, what the text says [21, 22].

The next step was to find meaning units, condense, and then encode them [21]. The codes were analyzed and systematized, and five main categories appeared, and the codes were sorted into these main categories. 

(1) Values and ethics. (2) The system. (3) Management. (4) Patient. (5) Nursing. 

The codes and the content in all the main categories were analyzed, condensed, and placed into subcategories, and a brief abstract was written summarizing the main content from the different subcategories. These abstracts were considered across, and further meaning compacting was done in order to systematize and further abbreviate the text. In this process we identified two main themes containing and presenting the results:

1. Values and reflection are important for the nurses,
2. time pressure and nursing frustrations.

### 2.3. Trustworthiness

The validity, or authenticity and trustworthiness of a study, is seen in two dimensions, internally and externally. Internal validity states what is true about it. Did we use the appropriate concepts and methods to study the phenomenon? External validity is about what is transferable. There is no knowledge applicable under all circumstances and for every objective [19]. Both the interviews and the analysis were carried out together with all the authors. This enabled us during the entire process to discuss and verify perceptions and conclusions which enhanced the internal validity of the study. The study was conducted with a homogeneous group, nurses, and special nurses without leader responsibility and with daily patient contact. If the study had been carried out with nurse managers in the group, the results might have been different. The study's preliminary findings have been presented for experienced nurses and other researchers who found the results recognizable. The external validity of the study could have been strengthened if corresponding studies had been carried out in several hospitals. The study was carried out in a medium sized hospital in Norway with nurses from different somatic and psychiatric units. It represents diversity both in terms of fields of study and nurses' previous experiences and can thus be transferable to other nurses in other hospitals.

### 2.4. Ethical Considerations

The project has been reported to the Norwegian Social Science Data Services (NSD).

The participants received verbal and written information about the study and agreed in writing to voluntary participate. The collected data material is anonymized and treated confidentially.

## 3. Results 

As earlier described, two main themes were identified in the analysis, and the results are presented with sub-themes under each main theme:

1. values and reflection are important for the nurses,
2. time pressure and nursing frustrations in daily work.

### 3.1. Values and Reflection Are Important for the Nurses

The nurses from all units describe how ethics, values, and reflection on values are of great importance for the quality in nursing. The informants see the need for values that are formulated in writing and the need for specific aims for their workplace. Yet they feel that the values are solidly rooted even though they are not formulated in writing. The informants from somatic units say that they are aware of when the values are absent in practice and that it is their task to speak up about undignified conditions.

*“It would have been fine if it was like that, that we all went along the same road with values at the bottom. I feel, and can see it among my colleagues, that these values are solidly rooted. It has surely to do with the training and what one sort of has learnt”*

*“I would say that there is often more focus on the hustle and bustle and lack of space, so one would like to be reminded of the values. One needs it even though it lies deeply in us”***

*The Values Vanish into Thin Air*. The informants say that being nurses they need to have basic values and that what is most important is to see the patient and to treat him with respect. The informants say that their work is based on the basic nursing values learned in their training and from colleagues with good values. The nurses in somatic units experience that values are repressed and may be neglected in their daily work due to time pressure, lack of resources, and great challenges. Nurses from somatic units prioritize helping patients in grave and acutely life-threatening conditions. With limited resources, this can result in undignified conditions for other patients whose illnesses are not life-threatening. These patients, who, for example, have to wait a long time without anyone keeping an eye on them, are given insufficient or unsatisfactory nursing. In these cases, the nurses feel that the job they are doing is not good enough.

*“It has often to do with resources, unfortunately. At times these values vanish into thin air when it is a matter of whether the patient breathes or does not breathe, and so the prioritizing becomes tough and at that point I cannot exactly manage to see these values, well, and even if they are there in the tiny moment when you are with the patient, I do believe that there are many of us who think that we could have done a better job”***

*The Quality Varies*. The nurses in the somatic units experience the quality in nursing as varying on account of lacking resources and time pressure in their daily work. The informants state that the quality is good when patients' basic needs are taken care of, when they do not have to lie in the corridor but are given a room and when one has time to talk with them and their families. If the nurse finds time to do this “little extra” for the patient and does not have to run from room to room, she will feel that she has done a good job which will make her feel good inside.

The informants from the psychiatric units report that good quality in nursing depends on evidence-based knowledge and a flexible and individually adjusted program for psychiatric treatment, with client participation. This improves the quality and is respectful of the patient. The informants tell us that the quality is totally dependent on the composition of the staff and that experienced nurses can help to create an atmosphere of quiet and security in the milieu and also, for example, in a coerced treatment situation.

*“It is about offering flexible psychiatric treatment. That's what we are preoccupied with, the fact that no one is alike”*

The informants from somatic units inform us that they will go to great lengths for quality and that they feel it is important to adapt nursing measures to the patient's needs rather than focusing on procedure. Nurses from all the units emphasize that procedures cannot be followed in all situations and that one cannot produce recipes or procedures for interpersonal relationships.

*“It has to do with interpersonal relationships. We cannot write it, provide a recipe or a procedure to be followed in all situations. Because things can really go wrong then, since every situation is unique and there are so many facets in what we do. Experience and basic values and ethical principles are required, and good child rearing, that is always welcome”***

*Safety Is Not Always There*. Respect, genuine presence, and good communication with patients and relatives generate safety and is described as decisive for quality as a whole. At times the informants from the somatic units experience insecurity and lack of control in their work situation. They feel that it all is haphazard and that they have to rely on sheer luck when there is a lack of both time and resources.

*“I'm thinking a little about it in terms of safety and procedures. One can of course say that when they are busy developing it, it is a form of safety, but it happens quite often that when I am working I feel that things are not safe. When it is too much for you to cope when there are so many you should give care to. And there can be long periods between each time you are with a patient, so there is not much safety to speak about”*

In psychiatry, safety was described as a safe workplace which will not be closed down, where there is enough staff, and the technical and administrative systems are functioning. A candid and honest environment where nothing is too stupid to say among colleagues will also provide a sense of safety.

*Time for Reflection and Supervision Is Vital*. All the nurses confirm that professional and ethical reflection is important for nursing professionals, but in the somatic units formal arenas and time for reflection are lacking. The informants in the somatic units often have informal talks about reflection with other nurses and physicians in the course of their workday but are continually interrupted because things are hectic and one certainly cannot stop the running of the ward.

*“Well, the things that we know would be good for the quality we do not get done in practice because one has to keep things going. We haven't got the time to stop and even if the hospital maybe has taken a patient's life, we do not have time for reflection and certainly not together with a surgeon”*

Only two of the informants from somatic units have been offered clinical nursing supervision. The nurses from the somatic units have requested clinical nursing supervision, but regular offers have neither been made nor has there been a tradition for it. The nurses say that one can ventilate ideas, get feedback, and have one's values with you to the supervision, and this they believe may turn you a better nurse.

*“I'm in clinical nursing supervision and that is very useful since there are many cases that touch one deeply. It is mostly a place for letting it all come out, and then you get feedback from the others about what they are thinking, because they too have often been in the same situation”*

The informants from the psychiatric units had experienced formal clinical nursing supervision. In addition, they described a culture and an organizing of the daily work which provided adequate time for reflection and supervision. The nurses from the psychiatric units say that it is important to be observant and available to new staff who need clinical supervision and that clinical supervision has been important and essential for their own choice to continue in this profession.

*“I am thinking about the report between the shifts, well, it becomes partly a kind of clinical supervision plus that when one is at work one keeps the discussions going”*

The nurses from the psychiatric units describe a reflective workday with discussions about right or wrong, values, and reflection on values in most situations. This seems to be an internalized way of working and thinking for these nurses. The nurses from the psychiatric units confront many ethical dilemmas and say that it is important and necessary that several people take part in these discussions.

*“I do not think we say that today we are evaluating values in our unit, but it goes on all the time. A lot of it in the staff room, but also at morning meetings, team meetings, where things are taken up and discussed whether this is right, or should we do it differently”*

### 3.2. Time Pressure and Nursing Frustrations in Daily Work

*Constant Guilty Conscience*. The informants from the somatic units are often feeling frustrated and worn out on account of considerable work and time pressure. Several of the informants admit to constantly having a guilty conscience. They say they always have to go to greater lengths and run faster and that this work pressure gives them a feeling of mass production and of having to calm things down in their daily work which they feel are both unsatisfactory and tiring.

*“The pressure is huge, and there is quite simply too much to do. One does what one can, but it is perhaps not quite what one actually wants to do or manages to do. And one has a constant bad conscience, well, one feels that one should have done more and that's a strain”*

*Rock-Hard Priorities*. Some of the informants from the somatic units fear that they might turn into indifferent and superficial nurses because of the frequent rock-hard priorities given to basic needs in their daily work that as much as possible should be done in the shortest time. The great number of corridor patients also contributes to the fact that patients have to wait for a long time before the nurses can find time for them.

*“In my opinion there is rock-hard prioritizing in our daily work. First of all, one must prioritize the things that are most important for the patient, be it shaving, taking a shower or medicine, but then it has also to do with this great number of corridor patients, the corridors have actually been filled up, and then there are patients with newly discovered cancer diagnoses who have to lie out there in the corridor. One does not feel like going home and knowing that this is where she will be lying tonight, and … she is 50 years old and has pulmonary cancer. That's not good. Even if the patient seems to feel alright about it, it is clear that she only does so because she has cancer after all and that is much worse than lying in the corridor”*

*“The ethical values are actually challenged according to the way our employer organizes us within the existing staff resources. When there is sickness during the day one mustn't call anyone for the first one who is sick, but we are then supposed to go to greater lengths, run faster, we have to be everywhere at once and that is at the expense of safety and quality and care and …”*

The informants from the surgical units report that patients with cancer or chronic wounds have to wait because the picking up and bringing of patients to surgery are prioritized, and this is experienced by nurses as an ethically difficult prioritizing. The informants from the somatic units speak about the great gap between theory and practice, and they feel frustrated when reality is so far from the ideals they were taught in their training. The nurses also state that these conditions affect their professional pride and that they are discouraged and feel that they barely can keep their heads above water.

*“I feel like taking care of the poor person who was admitted to our hospital six months ago and is getting more and more depressed. At the same time you have this pressure all the time. You have to go to the surgery. You must pick up someone from post-op. All the time, while you are standing there confused, you actually want to go to that patient but you did get that phone-call. This burdens you with a very guilty conscience. I can never manage to do what I ought to do with the cancer patients. No, it's such a rush all the time. I feel that the management does not understand. They see all the names on the list but they do not quite understand what it feels like to be in the centre of the anthill”*

When time pressure became the topic in the psychiatric units, the informants pointed out that in psychiatry there should be sufficient time. In psychiatry, time pressure is incompatible with the basic values and principles in nursing and treatment. The nurses claim that a possible demand from the management for greater efficiency is not consistent with the way they are working.

*“It is all right to be busy, but never so busy that you feel that you are now losing your grip on someone. On the whole we have plenty of time, yes. We need that in psychiatry because we cannot go fast here, that is obvious. It is obvious that if the management comes with demands for far greater efficiency, then it will go all wrong, there will be a collision”***

*The Managers do not Understand What Is Happening*. The nurses from somatic units experience varying support and understanding from the managers. They feel that they do not have a say in decisions, are not being heard, and know that they would have much to contribute with if they were permitted to do so.

*“We would have welcomed being permitted to take some part in the decisions. Because it feels like we have no influence on anything, it would have been absolutely fantastic if we were permitted to take part and speak up about some matters. I do not believe they are aware of what we are doing during the day. We are just supposed to run faster and faster and keep our mouths shut. I feel that there won't be much quality in the long run, I really do”*

The nurses from somatic units describe how the management does not set aside sufficient time for talking about values or focusing on them, for example, in staff meetings.

*“The leaders are especially responsible for creating targets in their units and what should be focused on… but as said before, we are all responsible for supporting and complying with it”***

*The System Cannot Be Changed*. The informants from the somatic units place the responsibility for present conditions on the system without defining more specifically who or what the system is. The nurses say that, when the system does not function, it is to the patient's detriment. The system and its financial limitations are stated as the reason why ethical values are challenged by structural issues. The same system causes difficulties in terms of bringing about improvements.

One has the impression that the system is almost impossible to change or do anything about.

*“We talk about quality and about what we can do about it, like with procedures, and deviations, but there is a lot in this system that has to do with quality that we cannot do anything about. Like economy, that there is too little funding and all that. We could certainly have sat in a group discussing values, how to put them to best use, but a lot has to do with having more staff in order to be able to include quality and safety in our work, and respect that we hopefully have in any case”*

## 4. Discussion

### 4.1. Values in Practice

All the nurses in this study experience that ethics, values, and reflecting on values are of great importance for the quality in nursing. This is consistent with research on ethical sensitivity and clinical competence in nursing. The experiential basis in nursing is the encounter with ethics and value phenomena. The ability to be compassionately affected and empathic is anchored in ethics, as the basis for value susceptibility and depth of intentionality and motivation behind the nurses' actions. Emotions are of importance for the normative demarcation between impartial justice and utility maximization on the one hand and compassion on the other [23]. Moral and ethical sensitivity has implications beyond what is relevant for moral judgemental power. Moral sensitivity is essential for generating relevant clinical knowledge [24].

The nurses in our study say that it becomes obvious to them when the ethical values are absent in practice and that it is their duty to speak up about unworthy conditions. The fact that they are aware of the lack of values and unworthy conditions confirms their moral and ethical sensitivity which again rests on sensibility and the ability to be accessible in human encounters [25].

Our study demonstrates that the nurses' ability to be accessible in human encounters in their work is threatened by time pressure and lack of staff. The nurses in our study give expression to this by stating that they fear becoming superficial and indifferent nurses because their work load is too heavy for satisfactory nursing. This is a challenge involving professional healthcare education, the attitude of the entire national health service, politics, and the financial structures which have an impact on this setting [25].

The youngest nurses in our study suffer the greatest reality shock. They witness that there is a big difference between theory and practice and this gives rise to frustration. It is consistent with research around this phenomenon which shows that reality shock is traumatic; it is a reaction in newly trained nurses who cannot practice what they have been trained to do and wish to do [26].

In the opinion of our informants, what is most important is to see the patient and have respect for him, and they consider this to be a fundamental nursing value which they learnt in their training and from colleagues who have good values. None of the nurses refer to or mention codes for professional ethics as a basis for values. This is consistent with European studies that show that nurses lack knowledge about ethical codes and do not use them to evaluate moral aspects in practice. Nurses rely on personal values and experiences [6].

### 4.2. Caritative Leadership Theory

Nurses from the somatic units state that there is little focus on ethical values on the part of the management, and they maintain that it is the management that is mainly responsible for what is brought into focus. Bondas [1] has done research on nursing management and taken part in the development of Caritative leadership theory. caritative leadership (CL) has been developed from a caring science perspective with the caritas motive as its point of departure, human love, and mercy, focused on ministering to the patient. CL strives for an effective and sustainable development of nursing and caring, with a holistic view on patients, relatives, and staff. Together with the staff, the leader creates a caring culture with the patient at its centre [1]. In this type of culture the ethical values must be included and focused on, both by staff and leaders. The nursing management's focus on human dignity, human love, and mercy may be set aside by the introduction of a market oriented leader style derived from industry and business [1]. In her research on first line nursing management Bondas found that these leaders, together with the staff, have to prepare the ground for nursing care. Preparing the ground for nursing care is a continuous process for the nursing leaders which is important for the unit's core function, for creating direction and quality in nursing, and with the patient as main focus [27].

In our study, the nurses in somatic units repeatedly describe a culture where it seems that the managers do not prepare the ground for nursing care but are concentrating on production objectives, cost savings, and effectiveness instead of on human dignity and compassion. This is illustrated by the fact that the nurses have to prioritize transport of remunerative elective surgery patients, that is, part of the hospital's production, rather than giving care to cancer patients and other patients who are not part of the same production.

The nurses themselves experience this as an ethically difficult prioritizing and as unsatisfactory nursing. They also experience varying support from the managers and that the management does not understand what is going on or what it feels like to be a nurse “right inside the anthill.” Such practice is not compatible with a caritative, nurturing culture which should prepare the ground for good nursing care in the best interest of the patient, relatives, and the nurse [1, 27].

### 4.3. Supervision and Reflection in Practice

The nurses from the somatic units have little opportunity to get formal clinical supervision or to reflect on their practice, not even after serious incidents. In most somatic units, there is no established practice of clinical nursing supervision. The nurses who do have this offer are extremely pleased with it; they find it useful, and say that it can turn you into a better nurse.

The nurses from the psychiatric units described a reflective workday where the ethical values and reflection on them were present in most situations. It was an internalized way of working which facilitated good conditions for the patients as well as for the nurses. The nurses in psychiatry said that they were caught up in many ethical dilemmas and that it was important and correct that several people took part in the discussion of these issues. Confronting the suffering of the depressed and mentally ill patient activates the nurses own feelings of pain, and they seek help from colleagues when they experience the relationship to the patient as painful. They need a forum which enables them to verbalize and explore their own feelings, where they are met with acceptance and response where feelings are set into a professional context [28]. The nurses from the psychiatric units had all had clinical nursing supervision, and their workday was organized in such a manner that there were time and room for constructive reflection on the practice. This contributed to an improvement of the practice and prevented the nurses from taking incidents and problems from their practice home with them. Some of the informants said that supervision had been decisive for their choice to continue in their profession. To be able to discuss feelings and experiences from the practice with other nurses will help the nurse to understand her own moral stance in terms of other nurses and the organization. Clinical supervision can have a positive effect on the nurses' experience of their own wellbeing in as much as psychic symptoms such that anxiety is reduced and the feeling of being in control grows [2]. Summarized research on clinical supervision demonstrates that clinical supervision can be both advantageous for professional practice and also for the patients. It is the health organizations who are responsible for developing and maintaining clinical supervision for nurses [29]. Clinical supervision is a reflective practice where nurses are given support and supervision on practice, which then will contribute to the nurses' professional and personal development [30]. 

### 4.4. Rock-Hard Prioritizations and Moral Distress

The nurses from the somatic units told about high work pressure, rock-hard prioritizations, varying quality, and sometimes a lack of safety. They experienced that the values “vanished into thin air,” that is, became insignificant in what they referred to as rock-hard prioritizations of basic needs. As a result of their work situation, the informants felt frustrated, worn-out, and feared becoming superficial. They describe a busy day with constant bad conscience and a feeling of not being adequate. Several studies from Norwegian hospitals and nursing homes show that lacking resources lead regularly to suboptimal professional standards for nursing and medical treatment [5]. The tendency is to lower the standard in order to safeguard the patient's vital needs, and this is at the expense of good practice and all-round care [31]. The nurses have to reduce their professional obligations and adapt to reality, and this creates a state in the nurses that can be understood as moral distress. The nurses in our study tell about crowded corridors and patients with newly diagnosed cancer who must lie in the corridor. This affects the nurses deeply. This is in accordance with studies showing that crowded rooms and corridors make it difficult to safeguard the patient's dignity and private life, and that the nurse feels moral unease for not being able to give the patient care in accordance with professional ideals [3]. Research on moral distress shows that it can arise on the basis of clinical situations and internal factors in the nurse, for example, a sense of powerlessness and lack of control over the situation, as well as external factors in the culture or organization, for example, inadequate staff [32].

Research shows that moral distress is linked to stress of conscience, while moral stress is seen in a more psychological perspective. Moral distress and related concepts are linked to moral and ethical sensitivity and the ethical climate which forms the basis for the nurse's moral freedom of action [33]. Moral distress is destructive for the staff's moral vigour and integrity. Compromises with ethical values or duties may have prolonged and negative effects causing the staff to become insensitive to the moral aspect of their work, or they may leave their profession [32]. The nurses in our study experienced that they did not have a say in the matter and that there was nothing to be done with the system which caused these difficult conditions. They describe a feeling of powerlessness and lack of hope in terms of being able to influence and improve the conditions. None of our informants talked about leaving the profession, but they were discouraged and felt that they were barely keeping their head above water. Research shows that the concern about nurses leaving their profession as a result of moral distress has created a greater awareness among health leaders and that the need for changes in the system is necessary as a partial response to the increasing level of moral distress in the health service [9, 11]. As shown before, the nurses in the psychiatric units had access to clinical nursing supervision and constructive professional reflection, and none of these expressed frustration or a guilty conscience as a result of time pressure and lacking resources. As shown before, reflective practice promotes moral practice and we therefore believe that formal clinical nursing supervision and reflection, in addition to changes in the system, will also promote the values in practice and thus contribute to a reduction in moral distress. Recent studies show that there is a high degree of moral distress among health professionals in many countries. This indicates an urgent need to consider the moral habitability of our practice environments. Health professionals themselves need to reaffirm the values and convictions that are important for their practice. The healthcare environment must be organized in a way that supports and promotes the health staff's moral considerations. If the healthcare environment is not seen as a moral community but rather as simulated market places, then the health staff's moral freedom of action will be reduced and make them more vulnerable to moral distress [34].

## 5. Conclusion

The results of the study demonstrate that all the nurses believe that ethical values are of decisive importance for the quality in nursing. Most important is respect for the patient. The nurses from the psychiatric units have a more reflective workday where the values are internalized in their work. They state that time pressure is incompatible with their values.

The nurses from the somatic units experience that the values are repressed and become meaningless words in a busy workday with time pressure and resource shortage. These nurses have to lower their professional standard, thus compromising their clinical nursing values and ideals. The nurses from somatic units are worn out and feel frustrated; they are constantly troubled by a guilty conscience and a sense of powerlessness. We understand this as an indication of moral distress. Financial and organizational structures contribute to the fact that the values are repressed in the nurses' practice and that this, together with insufficient time for clinical supervision and reflection, can be the basic reason for moral distress. We suggest a change of system based on the caritative leadership theory which may contribute to a combining of nursing and administration in such a way that it prevents suffering in the patient and will also further the development of nursing and care, humane and professional, on all levels in the organization [1]. We also consider formal and regular clinical nursing reflection and supervision as crucial for safeguarding moral practice and thus preventing the development of moral distress.

## References

[1] Bondas TE (2003). Caritative leadership. Ministering to the patients. *Nursing administration quarterly*.

[2] Bégat I, Ellefsen B, Severinsson E (2005). Nurses’ satisfaction with their work environment and the outcomes of clinical nursing supervision on nurses’ experiences of well-being—a Norwegian study. *Journal of Nursing Management*.

[3] Torjuul K, Sorlie V (2006). Nursing is different than medicine: ethical difficulties in the process of care in surgical units. *Journal of Advanced Nursing*.

[4] Førde R, Pedersen R, Nordtvedt P, Aasland OG (2006). Får eldreomsorgen nok ressurser/care for the elderly in Norway still suffers from lack of resources. *Tidsskrift for den Norske Laegeforening*.

[5] Nortvedt P, Pedersen R, Grøthe KH (2008). Clinical prioritisations of healthcare for the aged—professional roles. *Journal of Medical Ethics*.

[6] Tadd W, Clarke A, Lloyd L (2006). The value of nurses’ codes: European nurses’ views. *Nursing Ethics*.

[7] Epstein EG, Hamric AB (2009). Moral distress, moral residue, and the crescendo effect. *Journal of Clinical Ethics*.

[8] Hardingham LB (2004). Integrity and moral residue: nurses as participants in a moral community. *Nursing Philosophy*.

[9] Kälvemark S, Höglund AT, Hansson MG, Westerholm P, Arnetz B (2004). Living with conflicts-ethical dilemmas and moral distress in the health care system. *Social Science and Medicine*.

[10] Sporrong SK, Höglund AT, Arnetz B (2006). Measuring moral distress in pharmacy and clinical practice. *Nursing Ethics*.

[11] Pauly BM, Varcoe C, Storch J (2012). Framing the issues: moral distress in health care. *HEC Forum*.

[12] Cavaliere TA, Daly B, Dowling D, Montgomery K (2010). Moral distress in neonatal intensive care unit RNs. *Advances in Neonatal Care*.

[13] Corley MC, Minick P, Elswick RK, Jacobs M (2005). Nurse moral distress and ethical work environment. *Nursing Ethics*.

[14] Gutierrez KM (2005). Critical care nurses’ perceptions of and responses to moral distress. *Dimensions of Critical Care Nursing*.

[15] Wilkinson JM (1988). Moral distress in nursing practice: experience and effect. *Nursing Forum*.

[16] Krueger RA, Casey MA (2009). *Focus Groups: A Practical Guide for Applied Research*.

[17] Webb C, Kevern J (2001). Focus groups as a research method: a critique of some aspects of their use in nursing research. *Journal of Advanced Nursing*.

[18] Sørfonden W, Finstad HH (2000). Forskerliv og hverdagsliv i samme rom—et tilbakeblikk på erfaringer med metoden fokusgruppe. *Vård i Norden*.

[19] Malterud K (2003). *Kvalitative metoder i medisinsk forskning*.

[20] Thornquist E (2003). *Vitenskapsfilosofi og vitenskapsteori for helsefag*.

[21] Graneheim UH, Lundman B (2004). Qualitative content analysis in nursing research: concepts, procedures and measures to achieve trustworthiness. *Nurse Education Today*.

[22] Elo S, Kyngäs H (2008). The qualitative content analysis process. *Journal of Advanced Nursing*.

[23] Nortvedt P (1997). Vi skaper ikke verdiene, men holder dem oppe—et bidrag til sykepleiens verditeori. *Vård i Norden*.

[24] Nortvedt P (2001). Clinical sensitivity: the inseparability of ethical perceptiveness and clinical knowledge. *Scholarly Inquiry for Nursing Practice*.

[25] Nortvedt P (2008). Sensibility and clinical understanding. *Medicine, Health Care and Philosophy*.

[26] Kramer M, Orvik A (2004). 1974. *Organisatorisk kompetanse—i sykepleie og helsefaglig samarbeid*.

[27] Bondas T (2009). Preparing the air for nursing care: a grounded theory study of first line nurse managers. *Journal of Research in Nursing*.

[28] Sneltvedt T (2004). Å være i en relasjon der en opplever en annens smerte. *Vård i Norden*.

[29] Butterworth T, Bell L, Jackson C, Pajnkihar M (2007). Wicked spell or magic bullet? A review of the clinical supervision literature 2001–2007. *Nurse Education Today*.

[30] Bondas T (2010). Nursing leadership from the perspective of clinical group supervision: a paradoxical practice. *Journal of Nursing Management*.

[31] Halvorsen K, Førde R, Nortvedt P (2008). Professional challenges of bedside rationing in intensive care. *Nursing Ethics*.

[32] Hamric AB (2012). Empirical research on moral distress: issues, challenges, and opportunities. *HEC Forum*.

[33] Lützén K, Kvist BE (2012). Moral distress: a comparative analysis of theoretical understandings and inter-related concepts. *HEC Forum*.

[34] Austin W (2012). Moral distress and the contemporary plight of health professionals. *HEC Forum*.

